# Infrared Spectroscopy of Pollen Identifies Plant Species and Genus as Well as Environmental Conditions

**DOI:** 10.1371/journal.pone.0095417

**Published:** 2014-04-18

**Authors:** Boris Zimmermann, Achim Kohler

**Affiliations:** Department of Mathematical Sciences and Technology, Norwegian University of Life Sciences, Ås, Norway; UMass, United States of America

## Abstract

**Background:**

It is imperative to have reliable and timely methodologies for analysis and monitoring of seed plants in order to determine climate-related plant processes. Moreover, impact of environment on plant fitness is predominantly based on studies of female functions, while the contribution of male gametophytes is mostly ignored due to missing data on pollen quality. We explored the use of infrared spectroscopy of pollen for an inexpensive and rapid characterization of plants.

**Methodology:**

The study was based on measurement of pollen samples by two Fourier transform infrared techniques: single reflectance attenuated total reflectance and transmission measurement of sample pellets. The experimental set, with a total of 813 samples, included five pollination seasons and 300 different plant species belonging to all principal spermatophyte clades (conifers, monocotyledons, eudicots, and magnoliids).

**Results:**

The spectroscopic-based methodology enables detection of phylogenetic variations, including the separation of confamiliar and congeneric species. Furthermore, the methodology enables measurement of phenotypic plasticity by the detection of inter-annual variations within the populations. The spectral differences related to environment and taxonomy are interpreted biochemically, specifically variations of pollen lipids, proteins, carbohydrates, and sporopollenins. The study shows large variations of absolute content of nutrients for congenital species pollinating in the same environmental conditions. Moreover, clear correlation between carbohydrate-to-protein ratio and pollination strategy has been detected. Infrared spectral database with respect to biochemical variation among the range of species, climate and biogeography will significantly improve comprehension of plant-environment interactions, including impact of global climate change on plant communities.

## Introduction

Global climate change is already forcing plants to adapt, and it is foreseen that by the end of the century we can expect widespread disruption and extinction of species [1]–[3]. Spatial and temporal shifting, such as earlier onset of flowering and repositioning towards the poles, demonstrate widespread disruption in phenology of plants [4]–[6]. However, the projections of future changes are limited by incomplete theoretical understanding and insufficient data to improve ecological models [6], [7]. To determine climate-related plant adaptation it is imperative to improve plant phenotyping and monitoring of plant communities by a cost-efficient and rapid methodology. Moreover, knowledge on plant traits could lead to artificial selection and genetic manipulation of economically important plants in order to develop cultivars adapted to extreme environmental conditions [8], [9].

Seed plants (spermatophytes) comprise almost 90% of all plant species. As all land plants, they alternate between sporophyte generation that makes up what we perceive as the plant, and a gamethophyte, a microscopic reproductive organism. Traditional plant phenotyping is focused on sporophytes, and obtaining objective and reproducible results is still a challenge to researchers. The main sources of variations are genetic differences within and among populations, effects of the environment on traits (phenotypic plasticity), and traits change over the course of growth (ontogenetic drift) [10]–[12]. On the other hand, phenotyping of male gametophytes (i.e. pollen grains) is largely unexplored, given that characterization is predominantly based on morphological features while biochemical properties are regularly omitted.

Pollen studies can provide insight into plant-climate interactions [13], [14], but unfortunately the research has remained basically unchanged in the last hundred years. Current pollen studies are still confined on time-consuming visual measurement of pollen under microscope by a qualified specialist [15]. Biochemical characterization of pollen is important for one other reason: Impact of environment on plant fitness is almost exclusively based on studies of female functions, while the contribution of male gametophytes is mostly ignored. The latest finding, based on meta-analysis of 96 studies, suggests that measuring female function alone may misrepresent the effect of environmental factors on plant reproduction [16]. During the last three decades there have been numerous studies on so-called pollen competition hypothesis and the relationship between pollen and offspring performance [3], [17], [18]. The confirmation of the extensive overlap of the pollen transcriptome with that of sporophytes has provided further momentum that pollen selection can influence sporophytic fitness [19].

Initial spectroscopic studies clearly demonstrated that infrared (IR) spectra of pollen can be used for simple and rapid pollen analysis through straightforward correlation between spectra and biochemical composition [20]–[23]. Our previous study on 43 conifer species [23] revealed quite significant biochemical differences between closely related pollen species, which is rather unexpected since pollen morphologies of congeneric and confamiliar species seldom display substantial dissimilarities. In fact, due to negligible morphological differences between related species, pollen grains are rarely identified to the species level by microscopy, and some may have to be placed in groups of multiple genera or families. What is more, owing to natural hybridization and introgression, identification of the exact plant species is problematic even by means of genetic analysis [24].

Due to the small number of analyzed species and narrow scopes (studies of aeroallergens and honey) studies so far have failed to recognize the potential of Fourier-transform infrared (FT-IR) spectroscopy for plant phenotyping. Consequently, the studies have not provided the massive data needed for deducing genetic and evolutionary conclusions. To exploit IR spectra of pollen for plant characterization we have conducted a broad study by the FT-IR spectroscopic techniques. The effect of phylogenetic differences on pollen composition was assessed by measuring the massive sample set of 300 different species, belonging to 53 families in all principal spermatophyte clades (Table 1, and Table S1 in File S1). The study has covered five distinct pollination seasons (2009–2013), thus enabling assessment of environmental effects on pollen as well.

**Table 1:** List of analyzed plant taxa (see Table S1 for details).

CLADE	ORDER	FAMILY	No. of genera	No. of species
eudicots	*Fagales*	*Betulaceae*	5	13
		*Fagaceae*	2	13
		*Juglandaceae*	3	7
	*Malpighiales*	*Salicaceae*	2	5
		*Euphorbiaceae*	1	1
	*Lamiales*	*Oleaceae*	3	6
		*Plantaginaceae*	2	7
		*Scrophulariaceae*	1	1
		*Acanthaceae*	1	1
	*Solanales*	*Solanaceae*	4	5
	*Cucurbitales*	*Cucurbitaceae*	1	1
	*Proteales*	*Platanaceae*	1	2
	*Saxifragales*	*Altingiaceae*	1	2
		*Paeoniaceae*	1	2
	*Sapindales*	*Sapindaceae*	2	5
		*Anacardiaceae*	1	2
		*Rutaceae*	1	1
	*Dipsacales*	*Adoxaceae*	2	5
		*Dipsacaceae*	2	2
	*Buxales*	*Buxaceae*	1	2
	*Rosales*	*Rosaceae*	9	13
		*Urticaceae*	2	3
	*Ranunculales*	*Ranunculaceae*	7	13
		*Papaveraceae*	4	7
	*Fabales*	*Fabaceae*	1	1
	*Brassicales*	*Brassicaceae*	1	1
		*Tropaeolaceae*	1	1
	*Caryophyllales*	*Polygonaceae*	1	5
		*Portulacaceae*	1	1
	*Geraniales*	*Geraniaceae*	1	1
	*Asterales*	*Asteraceae*	3	5
		*Campanulaceae*	2	2
	*Malvales*	*Malvaceae*	3	4
		*Cistaceae*	1	1
magnoliids	*Magnoliales*	*Magnoliaceae*	2	2
monocots	*Poales*	*Cyperaceae*	4	15
		*Poaceae*	18	31
		*Juncaceae*	1	2
		*Typhaceae*	1	1
	*Asparagales*	*Hyacinthaceae*	2	2
		*Iridaceae*	2	19
		*Asphodelaceae*	1	1
		*Xanthorrhoeaceae*	5	11
		*Asparagaceae*	1	3
	*Liliales*	*Liliaceae*	2	10
	*Arales*	*Acoraceae*	1	1
	*Zingiberales*	*Cannaceae*	1	1
	*Arecales*	*Arecaceae*	1	1
conifers	*Pinales*	*Cupressaceae*	12	24
		*Taxaceae*	1	2
		*Cephalotaxaceae*	2	2
		*Pinaceae*	5	29
		*Podocarpaceae*	1	1
ginkos	*Ginkgoales*	*Ginkgoaceae*	1	1

## Materials and Methods

### Samples

Pollen samples were obtained through fieldwork in the publicly funded institutions in the Republic of Croatia: the Ruđer Bošković Institute and the University of Zagreb. The study is a part of government-funded research, and therefore has been conducted with the full cooperation of the administrations of the involved institutions. The field studies did not involve endangered or protected species.

Samples of pollen were collected at two facilities of the University of Zagreb; the Botanical Garden of the Faculty of Science and the Botanical Garden “Fran Kušan” of the Faculty of Pharmacy and Biochemistry. Additional samples of common wind-pollinated species were collected at the facilities of the Rudjer Boskovic Institute and the Faculty of Science of the University of Zagreb. All the locations are in the City of Zagreb. 813 samples were collected altogether, belonging to 300 plant species (Table S1 in File S1). Pollen samples were collected during five pollination seasons (2009–2013). The pollen samples were collected directly from plants at flowering time, either by shaking flowers (anemophilous species) or collecting mature anthers (entomophilous species). The samples were kept in paper bags at r.t. for 24 hours (together with anthers for entomophilous species), and afterwards transferred to vials and stored at −15°C; in general their IR spectra were recorded within 48 h after sampling.

For identification of basic biochemicals in pollen a set of model compounds that included lipids, carbohydrates and proteins was measured to correlate with high positive or negative values in the principal component analyses loadings plots. Spectra of crystal lipids and carbohydrates were recorded above their melting temperature, and again at r.t. after cooling to obtain spectrum of amorphous phase (liquid and/or glass phase). Tristearin (1,3-di(octadecanoyloxy)propan-2-yl octadecanoate), triolein (2,3-bis[[(Z)-octadec-9-enoyl]oxy]propyl (Z)-octadec-9-enoate), phosphatidistearoylcholine (1,2-distearoyl-rac-glycero-3-phosphocholine), phosphatidioleylcholine (1,2-dioleoyl-sn-glycero-3-phosphocholine), stearic acid (octadecanoic acid), oleic acid ((9Z)-octadec-9-enoic acid), cellulose, amylose, amylopectin, sucrose, trehalose, fructose, glucose, gluten, α-casein, β-casein, κ-casein were purchased from Merck (Darmstadt, Germany) and Sigma-Aldrich (St. Louis, United States), and used without further purification.

### Meteorological data

Meteorological data for the five-year period 2009–2013 were gathered by the Meteorological and hydrological institute of Croatia. The measurements of air temperature and humidity, barometric pressure, precipitation and insolation were obtained at Zagreb-Grič weather station. The station is approximately 0.9 km north of the Botanical Garden of the Faculty of Science, and 1.8 km south-east of the Botanical Garden of the Faculty of Pharmacy and Biochemistry, the Ruđer Bošković Institute and the Faculty of Science of the University of Zagreb.

### Infrared measurements

Macroscopic transmission and reflectance IR spectra were recorded with a resolution of 4 cm^−1^ using cosine apodization on an ABB Bomem (Quebec City, Canada) MB102 single-beam spectrometer, equipped with cesium iodide optics and deuterated triglycine sulphate (DTGS) detector.

The transmission spectra of all the samples were recorded by measuring potassium bromide (KBr) sample pellets. KBr pellets were prepared by mixing and grinding approx. 1 mg of a sample with approx. 100 mg of KBr using a pestle and mortar, a the created KBr matrix was then cold-pressed without degassing into a transparent disk. Three KBr pellets were prepared and recorded for each pollen sample. The IR spectra of the sample pellets were recorded with a total of 10 scans, and transmittance spectra thus obtained are termed raw spectra. A sample-free setup was used to obtain the spectral background.

The reflectance spectra of 503 pollen samples belonging to 294 different species were recorded by measuring samples with the single-reflection attenuated total reflectance (SR-ATR) accessory. The ATR IR spectra were recorded with a total of 30 scans using the horizontal SR-ATR diamond prism with 45° angle of incidence on a Specac (Slough, United Kingdom) Golden Gate ATR Mk II or a Specac High Temperature Golden Gate ATR Mk II. Each spectrum was recorded as the ratio of the sample spectrum to the spectrum of the empty ATR plate.

### Spectral pre-processing and data analysis

The FT-IR spectra obtained as transmission spectra of the KBr sample pellets and reflectance spectra with the ATR accessory were preprocessed prior to calibration: all spectra were smoothed or transformed to first or second derivative form by the Savitzky–Golay algorithm using a polynomial of degree two and a window size of 11 points (original (nonderivated) or first derivative) or 15 points (second derivative) in total, followed by normalization by multiplicative signal correction (MSC) [25] or extended multiplicative signal correction (EMSC model with linear and quadratic component) [26]. For transmission spectra the spectral region of 400 cm^−1^ to 1900 cm^−1^ was selected for data analysis, while for reflectance spectra the spectral region of 800 cm^−1^ to 1900 cm^−1^ was selected.

The data sets were as follows: a) transmission IR spectral data set of all plant taxa (300 species, three spectra per specie; second derivative and EMSC corrected spectra), b) transmission IR spectral data set of Fagales order (33 species, three spectra per specie; first derivative and EMSC corrected spectra), c) transmission IR spectral data set of *Chamaecyparis* genus (14 samples, three spectra per sample; second derivative and EMSC corrected spectra), d) transmission IR spectral data set of *Pinaceae* family (45 samples, three spectra per sample; nonderivated and EMSC corrected spectra), e) reflectance IR spectral data set of all plant taxa (294 species, three spectra per specie; nonderivated and MSC corrected spectra with weighting vector), and f) reflectance IR spectral data set of *Betulaceae* family (10 samples, three spectra per sample; first derivative and EMSC corrected spectra).

Pre-processed spectra were used to evaluate biochemical similarities between pollen samples by using the principal component analysis (PCA) and the hierarchical cluster analysis (HCA). In the principal component analysis the variance-covariance matrix is diagonalised converting the variables in a new set of linearly uncorrelated variables called the principal components, The principal components are sorted such that the first principal component accounts for the largest variance, the second principal component for the second largest variance and so on. In FTIR spectroscopy of biological samples the co-linearity between variables is very high, meaning that by a small set of principal components the main variation patterns can be summarized. HCA plots (dendrograms) were calculated with Euclidian distance measure and Ward distance clustering algorithm.

The estimation of relative content of pollen lipids for *Iris* (18 species), *Quercus* (12 species) and *Pinus* (15 species) genera, collected during the 2011 pollination season, was obtained on the EMSC and baseline corrected spectra (details in the Fig. S1 in File S1). The quantitative spectral analysis was based on the three vibrational bands: lipid band at ∼1740 cm^−1^, amide II band at ∼1545 cm^−1^, and sporopollenin band at 833 cm^−1^. The average transmission spectra (based on three measurements per species) were scaled to amide II band (with sporopollenin band serving as a double-check for scaling), followed by spectral deconvolution (multipeak fitting of Gaussian and Lorentzian curves), and application of Beer–Lambert law, i.e. the linear correlation of absorbance (area under the curve with the lipid band as a centre) and quantity. The same procedure was used for the estimation of inter-annual variations of pollen content for the 14 individuals during the 2011–2013 seasons (*Abies*: *A. cephalonica* and *A. Pinsapo*; *Picea*: *P. orientalis, P. asperata, P. pungens, P. omorika* and *P. chihuahuana*; *Pinus*: *P. banksiana, P. heldreichii, P. resinosa, P. tabuliformis, P. ponderosa, P. pungens, P. nigra, P. mugo* and *P. peuce*).

For the analysis of carbohydrate-to-protein ratio in pollen, the most dominant parts of the reflectance spectra were downweighted by applying weighting vector that facilitated stable baseline in all spectra (details in Fig. S2 in File S1). For the analysis of pollination strategy the following taxa were denoted: 1) anemophilous: *Fagales* (except *Fagaceae*), *Pinales* (except *Podocarpaceae*), *Poales*, *Proteales*, *Anacardiaceae*, *Asteraceae (except Taraxacum)*, *Polygonaceae*, *Urticaceae*, *Plantago*; 2) entomophilous: *Asparagales*, *Brassicales*, *Cucurbitales*, *Dipsacales*, *Fabales*, *Geraniales*, *Liliales*, *Magnoliales*, *Malvales*, *Ranunculales*, *Sapindales* (except *Anacardiaceae*), *Solanales*, *Zingiberales*, *Acanthaceae*, *Campanulaceae*, *Paeoniaceae*, *Portulacaceae*, *Rosaceae*, *Scrophulariaceae*, *Digitalis, Taraxacum*; 3) double-strategy: *Arales*, *Arecales*, *Buxales*, *Malpighiales*, *Altingiaceae*, *Fagaceae*, *Ginkgoaceae*, *Oleaceae*, *Podocarpaceae*.

The spectral pre-processing and data analyses were performed by algorithms written for the setting of Matlab, V. 7.10 (The Mathworks Inc., Natick, United States).

## Results

### Overall assessment of pollen FTIR spectra

Roughly, the IR spectrum of pollen can be divided into specific regions containing signatures of lipids, proteins, carbohydrates and grain wall biopolymers called sporopollenins. As features related to these compounds are responsible for the lion's share of phenotypic attributes, FT-IR spectroscopy is excellent tool for biochemical analysis of pollen.

**Figure 1:**
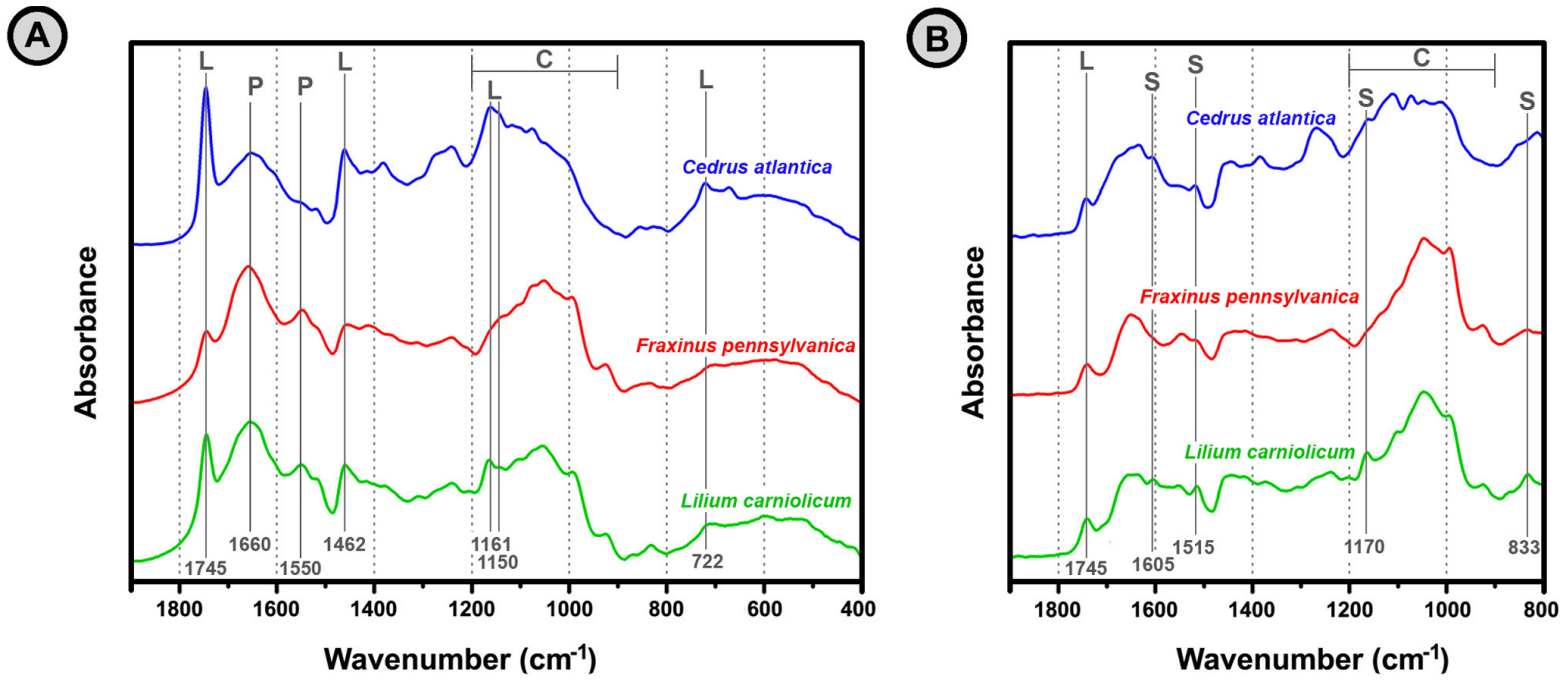
Infrared spectra of pollen. EMSC corrected transmission (**A**) and reflectance (**B**) IR spectra of *Cedrus atlantica* (Atlas cedar, Pinales order, *Pinaceae* family), *Fraxinus pennsylvanica* (Red ash; Lamiales order, *Oleaceae* family), and *Lilium carniolicum* (Carniolan lily; Liliales order, *Liliaceae* family) pollen. For better viewing the spectra are offset; the marked vibrational bands are associated with lipids (L), proteins (P), sporopollenins (S) and carbohydrates (C).

The typical spectra of conifer, monocot and eudicot pollens are shown in Fig. 1, along with vibrational band assignment of major biochemical components. The two main group of lipids, triglycerides and phospholipids, are characterized by the strong vibrational band at 1745 (C = O stretch), as well as by weaker bands at 1462 (CH_2_ deformation), 722 cm^−1^ (CH_2_ rocking), and 1200–1100 cm^−1^ spectral region (C–O stretch). In addition, phospholipids have two bands in 1200–1100 cm^−1^ spectral region (P = O stretch). Proteins are characterized by two strong and broad bands at 1650 (amide I: C = O st) and 1550 (amide II: NH deformation and C–N stretch). The vibrational bands associated with carbohydrates are predominant in 1200–900 cm^−1^ spectral region (C–O–C stretch, C–OH stretch, COH deformation, COC deformation, pyranose and furanose ring vibrations). The sporopollenin bands at 1605, 1515, 1171 and 833 cm^−1^ can be associated with the vibrations of aromatic rings. Although all pollens are composed of these same chemical building blocks the ratios of biochemicals can vary substantially, which is reflected in the corresponding IR spectra of pollens.

For measurement of a transmission spectrum a pollen sample was grinded and embedded into KBr matrix material in order to prepare a sample pellet. As a result, chemical components from a whole grain interacted with IR radiation. On the other hand, reflectance (ATR) measurements were based on intact pollen grains. It is important to note that radii of studied pollen grains varied from less than 10 µm to more than 50 µm. These grain radii are larger than the penetration depth of the IR light, which in reflectance measurement is in the 0.5–5 µm range. As a result, the grain wall was in greater interaction with the light than the grain interior. For that reason the obtained reflectance spectra have disproportional bias toward biochemicals present in pollen grain wall. Hence, while for smaller pollens (<15 µm radius) both transmission and reflectance techniques are resulting with comparable biochemical information, for larger pollen grains (>25 µm radius) the obtained spectra from these two techniques can be quite different (Fig. 1; see also Fig S3 in File S1 for differences between transmission and reflectance spectra).

One noticeable advantage of transmission measurement compared to reflectance is wider spectral range (up to 400 cm^−1^ in transmission and up to 800 cm^−1^ in reflectance spectra), which is due to different optical materials used in the two infrared techniques. However, since pollen spectra are devoid of strong absorption in the 800–400 cm^−1^ spectral region, in this regard the actual advantage of transmission measurement is negligible.

### Impact of spectral pre-processing on data analysis

Vibrational spectra of pollen are rich in information on biochemical constituents. However, different spectral bands can often be discriminated only after application of resolution-enhancement techniques, such as conversion of data into derivatives [27]. When conversion into derivatives is applied it is imperative that spectral features of biochemical constituents are well understood since this procedure will result with higher suppression of a broad signal than a narrow signal (providing that the signals are of equal amplitudes). Signal bandwidth in mid-IR spectroscopy, usually defined as full width at half-maximum (FWHM), can vary substantially, spanning approximately two orders of magnitude. Regarding the presented pollen spectra, the bandwidths of sporopollenin and lipid bands in 1800–1500 cm^−1^ region are 3–5 times smaller than the bandwidths of protein bands. Therefore, conversion of data into second derivative form will highlight spectral variations associated with lipids and sporopollenins. On the other hand, using the original (nonderivated) data will emphasize broad spectral features, such as protein bands in 1700–1500 cm^−1^ spectral region and carbohydrate bands in 1200–900 cm^−1^ region. Consequently, a complete set comprising original and derivative data has been used in the study in order to reduce risk that a substantial part of potentially valuable information is lost.

### Pollen composition related to taxonomy

**Figure 2:**
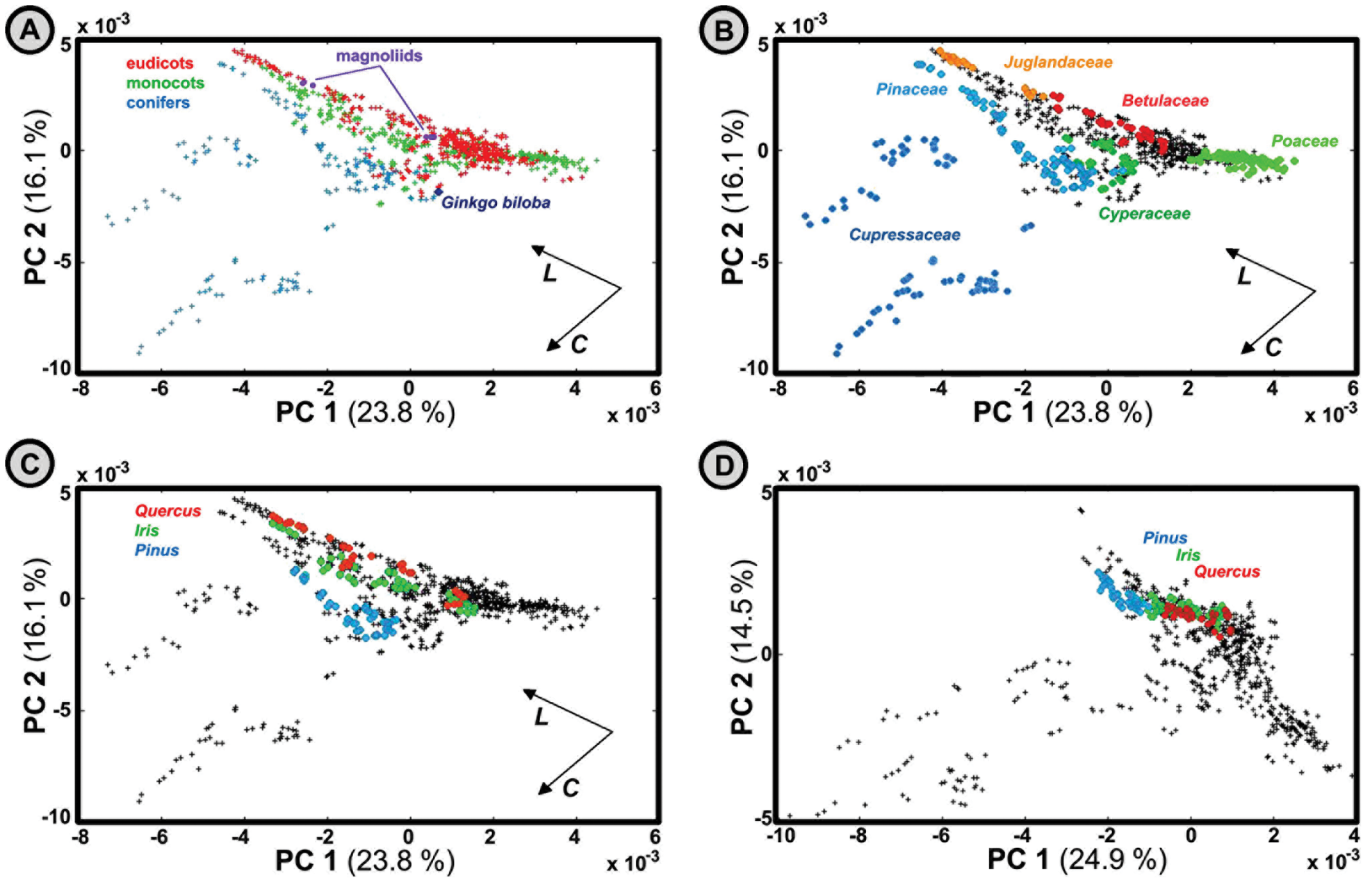
Correlation between spectroscopic data and taxonomy. PCA plots of transmission IR spectral data set (300 species, three spectra per specie; second derivative and EMSC corrected spectra) with depiction of: (**A**) eudicots, monocots, and conifers clades; (**B**) *Pinaceae*, *Cupressaceae*, *Poaceae*, *Cyperaceae*, *Betulaceae*, and *Juglandaceae* families; (**C**) *Pinus*, *Iris* and *Quercus* genera. The percent variances for the first five PCs are 23.83, 16.13, 11.30, 6.11, and 5.24. *L* and *C* vectors approximate the increase in relative amount of lipids and carbohydrates respectively (see Figs S3, S4 and S7 in File S1). (**D**) PCA plot of reduced data set, obtained by modelling spectral contribution of triglycerides (see Fig. S7 in File S1), with depiction of *Pinus*, *Iris* and *Quercus* genera. The percent variances for the first five PCs are 24.87, 14.48, 9.40, 7.17, and 5.91.

The extensive and diverse set of measured pollen samples belonging to all major spermatophyte clades has enabled assessment of similarities and differences between closely related species (congeneric and confamiliar), as well as between species that are very far related in historical relationship. The PCA of the second derivatives of transmission IR spectral data shows that the predominant spectral differences are the result of variations of bands associated with lipids, carbohydrates and sporopollenins (Fig. 2; see detailed PCA plots in the Fig S4 in File S1). The first four PC plots (as a function of wavenumber) have high positive or negative factor loadings associated with lipid bands (in PCs 1 and 2), carbohydrate bands (in PCs 1, 2 and 4), and sporopollenin bands (in PCs 3 and 4). Based on the PCA of the spectral data it is evident that the main biochemical differences arise due to the relative amount of lipids. Additional differences are caused by grain wall composition identified as sporopollenin to carbohydrate ratio. The detailed analysis of grain wall composition is presented in Fig. S3 in File S1. Considering related taxa, the analysis shows that spectral variability enables sufficient differentiation of plant families and confamiliar genera, and in a number of cases even congeneric species (Figs. S5 and S6 in File S1).

Clustering of spectral data belonging to phylogenetically related species is the predominant feature. However, it is also evident that pollen grains belonging to phylogenetically far-related plant clades can have similar relative biochemical composition. The PCA plot in Fig. 2A shows that all three principal clades of seed plants, i.e. conifers, monocots and eudicots, have the same general patterns of pollen composition. This includes large variations in the relative amount of lipids, present in all three clades, as well as smaller variations, that are mostly present in conifers samples, regarding the relative amount of carbohydrates.

Although, in general, the congenital species show clustering of spectral data, there are also noticeable exceptions. For example, the species belonging to *Iris*, *Quercus* and *Pinus* genera (as representative for monocots, eudicots and conifers) show extreme variations in the relative content of triglyceride lipids (Fig. 2C). Due to clear correlation of spectral features and biochemical constituents quantitative estimation of triglycerides in pollen have been obtained for congenital species of these three genera (Table 2, and Fig. S1 in File S1). The quantitative estimates reveal that pollen lipids can vary tenfold between minimal and maximal amount. While spectral variations between pollen groups of higher taxonomic levels (genera, families and orders) are associated with a number of different biochemical constituents, congenital variations are predominantly caused only by variations in the relative content of triglycerides. The spectral components associated with triglycerides can be extracted from the data set, in which case spectral variations for congenital species are greatly reduced resulting with enhanced PCA clustering of their spectral data (Fig. 2D). Therefore, it may be stated as a general rule that the congenital species show clustering of spectral data when spectral features of triglycerides are removed from pollen spectra. The detailed descriptions of estimation of pollen lipid content and transformation of spectral data by exctraction of triglyceride spectral features are provided in Figs. S1 and S7 in File S1.

**Table 2:** Relative lipid content (RLC) of pollen grains for *Pinus*, *Quercus* and *Iris* genera.

Genus	Species	RLC^a^
***Pinus***	*P. pinaster*	164
	*P. pinea*	147
	*P. ponderosa*	111
	*P. sylvestris*	**100**
	*P. nigra*	83
	*P. resinosa*	82
	*P. peuce*	79
	*P. strobus*	76
	*P. bungeana*	73
	*P. mugo*	73
	*P. tabuliformis*	69
	*P. heldreichii*	69
	*P. wallichiana*	59
	*P. densiflora*	58
	*P. banksiana*	57
***Quercus***	*Q. cerris*	401
	*Q. libani*	320
	*Q. frainetto*	283
	*Q. petreae*	172
	*Q. coccinea*	160
	*Q. faginea*	158
	*Q. pubescens*	157
	*Q. robur*	**100**
	*Q. rubra*	90
	*Q. coccifera*	63
	*Q. shumardii*	63
	*Q. ilex*	52
***Iris***	*I. pallida*	215
	*I. pseudopallida*	176
	*I. illyrica*	124
	*I. versicolor*	113
	*I. germanica*	**100**
	*I. sikkimensis*	78
	*I. bucharica*	77
	*I. spuria*	76
	*I. sanguinea*	75
	*I. unguicularis*	66
	*I. pseudacorus*	64
	*I. sibirica*	52
	*I. bulleyana*	50
	*I. orientalis*	39
	*I. japonica*	37
	*I. crocea*	33
	*I. aphylla*	33
	***I. graminea***	23

a The benchmark values of 100, calculated independently for Pinus, Quercus and Iris genera, is attributed to the mean value of lipid content of *Pinus sylvestris*, *Quercus robur* and *Iris germanica* pollen grains respectively (see File S1).

### Pollen composition related to pollination strategy

**Figure 3:**
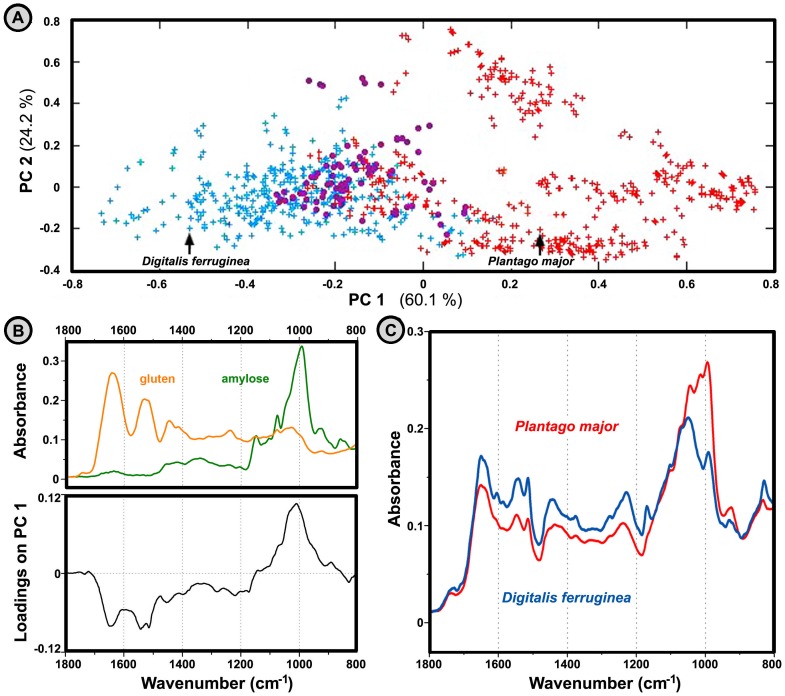
Correlation between spectroscopic data and pollination strategy (wind and/or insect pollination). (**A**) PCA plot of reflectance IR spectral data set (294 species, three spectra per specie; MSC corrected spectra) with depiction of entomophilous (120 species, blue), anemophilous (143 species, red), and double-strategy (31 species, purple) species. The percent variances for the first five PCs are 60.12, 24.19, 3.90, 2.86, and 1.51. (**B**) Reflectance IR spectra of carbohydrate amylose (green) and protein gluten (orange), and loadings plot on the first principal component of the PCA. (**C**) MSC corrected reflectance spectra of the two related species belonging to *Plantaginaceae* family: *Plantago major* (entomophilous specie) and *Digitalis ferruginea* (anemophilous specie).

As stated previously, the choice of pre-processing method can enhance or supress certain biochemical signals. When analysis of the reflectance data is obtained on original (nonderivated) spectra the broad signals associated with proteins and carbohydrates are highlighted while sporopollenin and lipid signals are supressed. The resulting analysis shows that there is an almost clear spectral separation between anemophilous (wind pollinated) and entomophilous (insect pollinated) species based on the overall ratio of proteins and carbohydrates (Figs. 3). Compared with entomophilous plants, anemophilous plants have increased relative content of carbohydrates.

### Spectroscopic fingerprinting of pollen

**Figure 4:**
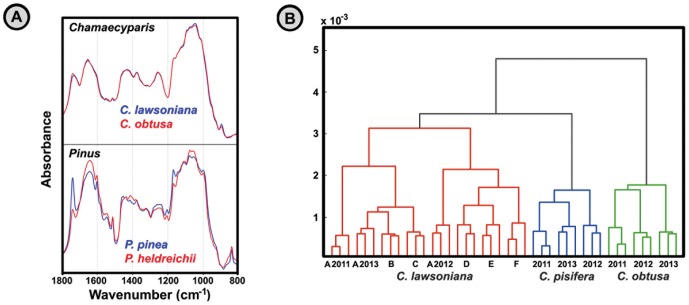
Identification of pollen based on their IR spectra. (**A**) Transmittance (KBr pellets) spectra of pollen belonging to *Chamaecyparis* and *Pinus* species (EMSC corrected spectra). (**B**) HCA of transmittance spectra belonging to *Chamaecyparis* samples (three spectra per sample; second derivative and EMSC corrected spectra). *Chamaecyparis lawsoniana* cultivars: A ‘Triomf van Boskoop’, B ‘Kelleriis Gold’, C ‘Alumigold’, D ‘Columnaris’, E ‘Silver Queen’, F ‘Tharendtensis Caesia’.

The identification of congenital species was conducted for species of *Chamaecyparis* that show minute spectral differences: *C. obtusa*, *C. pisifera* and *C. lawsoniana*. Fig. 4A shows that these spectral differences are much more subtle than is the case with congenital species of *Pinus* genus. The testing set comprised pollen samples belonging to one individual each of *C. obtuse* and *C. pisifera*, as well as six different cultivars of *C. lawsoniana*. Moreover, the pollen samples belonging to one *C. lawsoniana* cultivar and the two *C. obtuse* and *C. pisifera* individuals were collected and measured during the three consecutive pollination seasons (2011–2013). As can be seen by the result of cluster analysis in Fig. 4B, the three *Chamaecyparis* species are easily differentiated by their characteristic IR spectra. Although *C. lawsoniana* samples comprised different cultivars with substantial genetic differences, nevertheless all the samples are well separated in one cluster from the other species.

### Annual and seasonal variability

**Figure 5:**
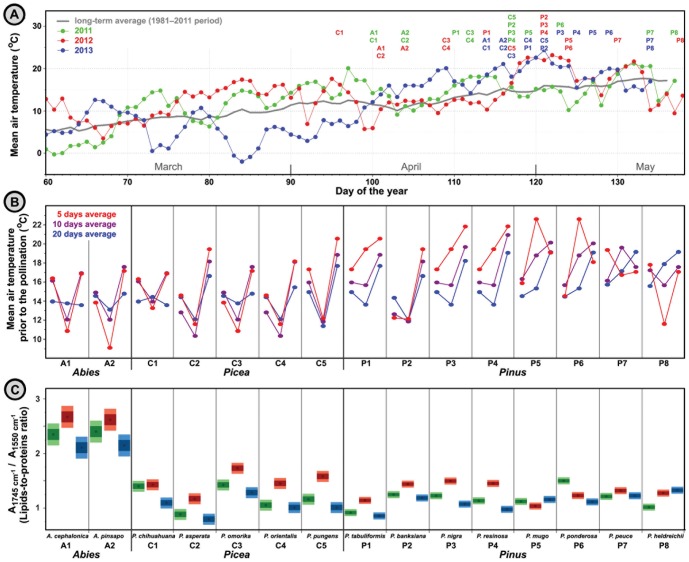
Correlation between spectroscopic and climate data. (**A**) Mean air temperature for Zagreb (Grič weather station) during 2011, 2012 and 2013 seasons. Pollination onsets of *Pinaceae* species are indicated with symbols (A: *Abies*, C: *Picea*, P: *Pinus*). (**B**) Mean air temperature 5, 10 and 20 days prior to the pollination start date. (**C**) Ratio of absorbance at 1745 and 1550 cm^−1^ in the transmittance spectra (KBr pellets) of pollen belonging to *Pinaceae* samples in 2011, 2012 and 2013 seasons (three spectra per specie; EMSC corrected spectra). Dark and light areas indicate ±1 and ±2 sample standard deviations respectively; standard deviations were determined for each genus separately, and were based on three spectra per sample (nine spectra per species).

In order to monitor inter-seasonal variations of pollen content, 15 individuals, each belonging to different species of *Pinaceae* family, were measured during the three seasons: 2011, 2012 and 2013 (Fig. 5). The spring pollination periods during the three seasons were warmer than the long-term average (Fig. 5A). The pollination season for the sampled *Pinaceae* species was postponed in 2013 for approximately 15 days due to cold and extremely rainy weather. This cold period was followed immediately by spike of warm weather.

The IR spectra show large inter-annual variations in pollen composition of the measured *Pinaceae* individuals. These variations are predominantly connected to lipid content, as indicated by variations in band at 1745 cm^−1^. Since the other principal biochemicals, such as carbohydrates and proteins, were quite invariant, the variations in lipid content have been plotted in Figure 5C as ratio of absorbance of the lipid band (1745 cm^−1^) to that of the protein band (1550 cm^−1^). Pollen composition of 2011 and 2013 samples is rather uniform, and in most cases is within the scope of measurement error (2–3 standard deviations). Pollen composition of 2012 samples deviates considerably from 2011 and 2013 values, in most cases more than 3–5 standard deviations. Moreover, the 11 individuals that have been pollinating first show the same pattern in lipids content: highest in 2012 and lowest in 2013. For example, for the four *Pinus* individuals (*P. tabulliformis*, *P. banksiana*, *P. nigra* and *P. resinosa*) the inter-annual variations in lipid content were remarkably uniform: increase in lipid content in 2012 was 24±2% (compared to 2011), while the decrease in 2013 was 9±1% (compared to 2011). This pattern is in good correlation with temperature profiles prior to the pollination start dates. Figure 5B shows that several weeks prior to pollination, during which the male cones (strobili) with pollen were being developed, the first 11 individuals were under influence of similar climate patterns: warmer weather in 2011 and 2013, and colder in 2012. The four *Pinus* individuals (*P. mugo*, *P. ponderosa*, *P. peuce* and *P. heldreichii*), that deviate from other 11 individuals in the inter-annual variations of pollen composition, were under influence of different climate patterns.

## Discussion

The characterization of seed plants by FT-IR of pollen has several favourable qualities. The desiccated nature of grains provides relatively stable biochemical composition, and thus enables simple manipulation and measurement of pollen. Moreover, our results identify biochemical variability of pollen grains at different taxonomical levels; genera, family, order and higher clades. The measurement on *Chamaecyparis* genus samples clearly indicates that spectral variation within species is smaller than the differences between species. This is particularly true when spectral variability associated with triglyceride content is extracted from the data set and disregarded (Fig. 2D). Regarding plasticity, the present study has addressed inter-annual variations in pollen content of samples belonging to the same parent plants. These variations are predominantly expressed as variations in pollen nutrient content in the form of lipids.

The details on IR methodology, its benefits and constraints, are discussed first. However, the impact of novel findings on plant biology studies is discussed as well, since this study provides the first comparative analysis of pollen biochemical composition.

### Method overview

The presence of unique features in an IR spectrum that correspond to the specific molecular structure is a major reason for broad acceptance of FT-IR spectroscopy for definitive material identification and chemical confirmation. As an analytical tool IR spectroscopic techniques have desirable features, including precision, speed, ease-of-use, minimal or no sample preparation, and the avoidance of sample destruction. The findings presented here are a direct result of conducting study on large and diverse set of plant taxa, and therefore emphasize benefits of low-cost, high throughput and high-dimensional methodology.

Traditional biochemical analysis of pollen is done by chemical pre-treatment followed by chromatography [28], [29]. The highly resistant grain walls and an unusual composition with low water content presents severe obstacle for routine analysis of numerous pollen samples. The contemporary pollen analysis is based on transmission electron microscopy of chemically pre-treated samples, and it offers a visual picture of organelles of the pollen grain [30]. Both approaches are time-consuming, expensive and alter the pollen sample in the pre-treatment process. As opposed to traditional methods, FT-IR spectroscopy is a non-destructive analytical technique that enables measurement of samples as found in nature. Moreover, the direct costs of measurements (including consumables and working hours) are significantly reduced by avoiding chemical pre-treatment. For example, in our study the average time of IR data gathering per sample (including sample preparation and spectral acquisition) was less than three minutes. Finally, IR spectroscopy yields similar, but complementary, information to Raman spectroscopy, a vibrational spectroscopy method that has seen extensive use lately in pollen research [23], [31]–[33].

The significant problems in infrared measurement of biological samples, such as pollen, are spectral reproducibility and separation of informative signals from non-informative ones. The measured spectra can show strong scatter effects and baseline problems resulting in an inferior spectral reproducibility. These problems, especially present in the transmittance spectra, were addressed by differentiation (transformation into first and second derivatives) and multiplicative signal correction (MSC and EMSC preprocessing). These automated preprocessing methodologies, with high level of standardization, inhibit data errors attributed to instrumentation or personal bias. It should be pointed out that FT-IR spectroscopy offers high measurement reproducibility, especially compared to traditional methods where complex chemical pretreatment, sample separation and human-based analysis leads to considerable intra-sample variability.

As shown previously, the IR spectoscopy of pollen enables identification of biochemical constituents. It should be emphasized that the general feature of an IR spectrum of pollen is the result of a relative content of structural biochemicals, specifically lipids (triglycerides and phospholipids), carbohydrates (starch and cellulose), sporopollenins, and a variety of proteins. The so-called coding and functional biochemicals, such as nucleic acids and enzymes, are present in pollen in significantly lesser amount than structural biochemicals, and thus constitute minor features of an IR spectrum. As a result, FT-IR measurement of biochemical composition of pollen offers complementary information that is overlooked by genomic and proteomic methods.

PCA plots in Figs. 2, 3, S5 and S6 show excellent potential of IR spectral data for various discriminative analyses, such as for taxonomy-based supervised classification as demonstrated previously by our study [23]. Moreover, the methodology enables numerical analysis and classification of pollen composition in the original spectral form or as latent variables, such as PCA loadings and scores. A spectral component of a biochemical class can be extracted from the data set in order to create partial biochemical classifier, as was demonstrated for triglycerides in Fig. 2D. In addition, a more precise quantitative estimation of a specific biochemicals can be obtained by signal fitting for congenital species, as demonstrated for triglycerides in pollen belonging to *Iris*, *Quercus* and *Pinus* genera (Table 2). Finally, different pre-processing methodologies can provide different information regarding biochemical constituents, such as putting emphasis on narrow sporopollenin bands or broad protein bands by using derivative or original spectra respectively.

FT-IR spectroscopy lacks qualitative precision of the traditional cytochemical analysis, that can separate sample into individual components (for example with extraction, chromatography and electrophoresis). However, it can provide rapid and economical comparative analysis of a large number of samples with definitive identification of main groups of biochemicals. Specifically, our study has interpreted biochemically the spectral differences related to environment and to taxonomy: 1) large variations of absolute content of triglycerides for congenital species pollinating in the same environmental conditions, indicating the effect of genetic variations on pollen content (Table 2 and Fig. 2); 2) noticeable inter-annual variations of pollen trygliceride content for the same parent plants (including clear correlation with climate data), indicating the effect of phenotypic plasticity on pollen content (Fig. 5); 3) clear correlation between carbohydrate-to-protein ratio and pollination strategy (Fig. 3); and 4) clear correlation between cellulose-to-sporopollenin ratio and taxonomy (Fig S3 in File S1). It should be emphasized that currently there is no benchmark method for practical assessment and comparative study of pollen composition, due to previously stated experimental difficulties. Therefore, here introduced FT-IR spectroscopy offers, not only simple, fast and reliable plant characterization, but it also enables unique novel insight on plant biochemistry, ecology and evolution.

The presented FT-IR methodology has ample advantages for rapid worldwide implementation: 1) Available infrastructure and expertise due to widespread use of FT-IR techniques, even in developing countries; 2) Simple, rapid and economical measurement, devoid of any chemical pre-treatment; 3) Semi-automated process of data collection and analysis; 4) Avoidance of specific high-skilled expertise, as opposed to the traditional morphology-based pollen studies; and 5) Attainable remote analysis of pollen spectra due to complete digitalization of data.

### Pollen characterization: phylogenetic variations

In general, the related species have smaller biochemical differences than the unrelated ones, most likely as a result of adaptation of the whole clade to relatively restricted environmental conditions. Consequently, for the related species the analyses show clear clustering of spectral data within rather narrow range, as can be seen for example in Fig. 2B for *Poaceae* and *Cyperaceae* pollen data. This property enables rapid identification of the exact family or genera of an unknown pollen sample, as was previously demonstrated for small sample set [23]. The somewhat larger variations measured in conifers primarily affect *Cupressaceae* pollen samples. Probable cause for outlying spectral features of *Cupressaceae* samples is unusually thick cellulose part of the grain wall (intine) (Fig. 2B, and Fig. S3 in File S1). The most remarkable feature, however, is similar relative biochemical composition of pollen grains belonging to phylogenetically far-related plant clades, as shown in Fig. 2A for all three principal clades of seed plants. The same general pattern of pollen composition in conifers, monocots and eudicots is predominantly due to large variations in relative amount of lipids.

Although the application of pollen IR spectra for identification of congenital species has been examined previously [21], [22], our study of *Chamaecyparis* species was innovative by taking into consideration the substantial genetic and inter-seasonal variations within species. The finding on *Chamaecyparis* sample set is in agreement with the differentiation of congeneric *Corylus* species (Fig S6 in File S1). This clearly indicates that spectral variation within species is smaller than the differences between species. In addition, it is clear that pollen composition varies to some extent due to seasonal conditions. While the inter-seasonal variations are not impairing the identification of species, it is however detrimental for sub-species identification of cultivars.

Of particular interest are samples belonging to congenital species that have considerably varied natural habitats as a result of divergent adaptations. For example, the species belonging to *Iris*, *Quercus* and *Pinus* genera are widely distributed throughout the Northern Hemisphere, ranging from cold latitudes to tropical. Our data shows that the predominant spectral variation in the all three genera is caused by enhanced content of triglycerides (Fig. 2C, Fig. S7 in File S1, and Table 2). The extreme variation in relative lipids content observed for these congenital species is remarkable regarding that these individuals have been growing in the same environment (within 100 m radii from each other). For example, the difference in absolute lipids content between *Quercus cerris* (on the higher end) and *Quercus ilex* (on the lower end) is almost tenfold. This is highly unexpected regarding the almost identical morphology, and especially concerning that *Quercus* genus lacks effective genetic isolation that results with frequent hybridization [24], [34].

### Pollen characterization: phenotypic plasticity

The inter-annual variations in content of *Pinaceae* pollen samples demonstrate the influence of climate conditions on pollen lipids (Fig 5). The interesting finding is the apparent negative correlation between lipids content and temperature, where colder weather induces higher production of lipids in pollen. Since pollen lipids serve as carbon and energy reserve, it is plausible that an increased storage of pollen lipids is a coping mechanism for countering adverse effects of colder seasons.

The disruption in flowering patterns in 2013 of the monitored *Pinaceae* individuals, caused by prolonged cold and rainy season, had resulted with surprisingly little effect on pollen composition. The pollen composition in 2011 and 2013 show considerable resemblance, although the pollination start dates in 2013 were shifted for some two weeks. On the other hand, the pollen composition in 2012 shows large deviation from the composition obtained in 2011, although the flowering phenology in the two seasons was quite invariant. Therefore, it is probable that temperature conditions prior to pollination have predominant influence on nutrient composition of pollen. This notion is in agreement with the recent finding that heatwaves have stronger impacts on physiological processes than changing mean climate [6].

### Impact of IR characterization of pollen on plant biology and ecology

Reproductive strategy and allocation of resources regarding plant fitness is traditionally researched from the perspective of female functions. Even when male functions are taken into consideration this is almost entirely based on reproduction traits associated with sporophyte generation, such as per-plant pollen production. In contrast, exclusive male traits, such as quality of pollen grains, are mostly overlooked. The traditional modelling of pollen dispersal assumes that mating probability is predominantly affected by distance between potential mating partners. However, recent studies of plant parentage assignment, obtained by DNA microsatellites, have shown that distance explains only a portion of the variation in mating patterns, and often a relatively modest portion [35]. In fact, long-distance pollination is common in anemophilous as well in entomophilous plants. Mean pollination distances are well above mean nearest neighbour distances and possibly even in a range of a few kilometres. The study of an extreme isolated population of *Pinus sylvestris* has shown that even when a nearest population is tens of kilometres apart, pollination with immigrant pollen is still present [36]. Concerning that single tree can produce millions of pollen grains [37] that could be wind transported for tens of kilometres, it can be expected that individuals producing pollen with increased nutrient content could rapidly propagate these traits throughout a population. As a result, accurate measurement of pollen biochemical composition by IR spectroscopy should offer unique new perspective on reproductive success and total plant fitness.

Our study has revealed one remarkable feature of pollen composition: An extensive variability of pollen triglyceride content. Pollen triglycerides primarily serve as carbon and long-term energy reserves in the form of intracellular spherical droplets that are termed lipid bodies [28], [29]. For a long time carbohydrates were considered principal nutrient reserves in mature pollen and were, therefore, extensively studied [38], as opposed to pollen lipid reserves that gained less interest. However, recent insights reveal that the role of pollen lipids is more important than previously acknowledged [39], especially considering that in some species, such as *Olea europea*, the lipid bodies are the only nutrient reserves present in the mature pollen [40]. More importantly, pollen lipids have a crucial role in germination and pollen tube growth [40]. This is of significance from evolutionary standpoint since pollen tube growth is the principal trait in the pollen competition hypothesis. Our findings, that lipid content of pollen is influenced by both genetic and environmental variations, support this hypothesis. It can be assumed that high triglyceride nutrient reserves in pollen, such as those measured in several *Qurcus*, *Pinus* and *Iris* species (Table 2), play a decisive role during pollination, with a significant effect on pollen siring success. Otherwise the unnecessary waste of valuable biochemicals would quickly result with the elimination of the underlying genetic cause from a population.

Another interesting result of the IR study is the correlation between pollination strategy and the protein-to-carbohydrate ratio. Although it is too soon to conclude if this is anything more than a coincidental result, the finding is supported by the IR spectral features of species showing a double pollination strategy, such as *Quercus* and *Fraxinus* [41], that display intermediate spectral features. A possible explanation for increased relative content of carbohydrates in anemophilous plants is the vital function of cytoplasmic saccharides in pollen resistance to dehydration. The saccharides, principally starch and sucrose, act as membrane stabilizers [38], [42], and could enable prolonged viability of anemophilous pollen during its airborne transport. Nevertheless, the possible crucial role of cellulose, present in the intine part of the grain wall, cannot be excluded.

## Conclusion

The novel findings regarding large variations in nutrient content of pollen, especially in the form of triglycerides, imply that male functions and pollen competition should be incorporated into ecological and evolutionary studies. Biochemically based spectral data enable not only more thorough analysis of pollen than grain morphology by microscopy, but also enable simple, rapid and economic assessment of plant-environment interactions. Moreover, biochemical characterization of pollen can be a valuable tool for trait-based approach in community ecology.

The infrared spectral data covered by this preliminary study already surpass data on some more prominent plant traits [10]. Development of standardized database of pollen IR spectra would allow rapid expansion of worldwide plant data by the global scientific community, and would serve as a starting point for identification, classification, biochemical characterization and general data mining. Implementation of the global spectral database covering a range of plant samples, with different taxonomical, temporal and spatial origin, should lead to a novel understanding of the plant-environment interactions.

## Supporting Information

File S1 Included in File S1: Figure S1. Estimation of pollen lipid content. Figure S2. Spectral pre-processing for estimation of carbohydrate-to-protein ratio in pollen. Figure S3. Pollen grain wall composition of *Pinales* order. Figure S4. PCA of transmission IR spectral data set. Figure S5. PCA of *Fagales* order. Figure S6. PCA and HCA of *Betulacea* family.Figure S7. PCA with modelling spectral contribution of triglycerides. Table S1. List of analyzed pollen taxa.
(PDF)
