# Supplementary material for: Infrared Spectroscopy of Pollen Identifies Plant Species and Genus as Well as Environmental Conditions
Source: PLoS One. 2014 Apr 18;9(4):e95417. doi: 10.1371/journal.pone.0095417 (PMC3991668; doi:10.1371/journal.pone.0095417)
Supplement: File S1 — Included in File S1: Figure S1. Estimation of pollen lipid content. Figure S2. Spectral pre-processing for estimation of carbohydrate-to-protein ratio in pollen. Figure S3. Pollen grain wall composition of Pinales order. Figure S4. PCA of transmission IR spectral data set. Figure S5. PCA of Fagales order. Figure S6. PCA and HCA of Betulacea family.Figure S7. PCA with modelling spectral contribution of triglycerides. Table S1. List of analyzed pollen taxa. (PDF) [file pone.0095417.s001.pdf]

Supporting Information for:

Infrared spectroscopy of pollen identifies plant species and genus as well as environmental conditions

Boris Zimmermann, **Achim Kohler**
Department of Mathematical Sciences and Technology, Faculty of Environmental Science and Technology, Norwegian University of Life Sciences, 1430 Ås, Norway.

<u>Table of Contents</u> <u>Page</u>

Estimation of pollen lipid content	2
Carbohydrate-to-protein ratio in pollen	3
Pollen grain wall composition	4
Correlation between spectroscopic data and taxonomy	5
Fagales order pollen samples	6
Triglycerides content of pollen	7
Table S1. List of analyzed pollen taxa	9

Estimation of pollen lipid content

The EMSC and baseline corrected spectra were used for the estimation of relative lipid content in pollen belonging to *Pinus*, *Quercus* and *Iris* genera. The analyses were based on the three vibrational bands: lipid band at ~1740 cm^{-1}, amide II band at ~1545 cm^{-1}, and sporopollenin band at 833 cm^{-1}. The average transmission spectra (based on three measurements per species) were scaled to amide II band (with sporopollenin band serving as a double-check for scaling), followed by spectral deconvolution (multipeak fitting of Gaussian and Lorentzian curves), and application of Beer–Lambert law, i.e. the linear correlation of absorbance (area under the curve with the lipid band as a centre) and quantity (Fig. S1). The obtained relative lipids content for the three genera are presented in Table 2.

The estimation of relative content of pollen lipids is based on the following approximations: 1) all congenital species have pollen grains with identical morphology, 2) sporopollenin and protein content in pollen for all congenital species is quantitatively invariant, 3) lipids content of pollen can be estimated by applying Beer–Lambert law on transmission spectra of the KBr sample pellets. Although the third approximation is quite far off due to diffuse reflection and saturated absorption, similar pollen morphology of congenital species will result in comparable deviations from Beer–Lambert law. The maximal values (Table 2) are probably underestimated regarding that saturated absorption is higher for samples with elevated lipids content. Different fitting parameters were assessed and the results typically varied approximately 10 % from the values reported in the Table 2.

Parameters used for spectral deconvolution:
For *Pinus* species: spectral range: 1900-1496 cm^{-1}; No. of curves: 6; Fitting constrains (function, FWHM, approx. position of peak value): Lorentzian (20, 1515 cm^{-1}), Lorentzian (50, 1545 cm^{-1}), Lorentzian (20, 1600 cm^{-1}), Gaussian (110, 1635 cm^{-1}), Gaussian (130, 1660 cm^{-1}), Lorentzian (25, 1745 cm^{-1}).
For *Quercus* species: spectral range: 1900-1481 cm^{-1}; No. of curves: 5; Fitting constrains (function, FWHM, approx. position of peak value): Lorentzian (50, 1510 cm^{-1}), Lorentzian (50, 1545 cm^{-1}), Gaussian (150, 1620 cm^{-1}), Gaussian (100, 1660 cm^{-1}), Lorentzian (25, 1745 cm^{-1}).
For *Iris* species: spectral range: 1900-1481 cm^{-1}; No. of curves: 5; Fitting constrains (function, FWHM, approx. position of peak value): Lorentzian (50, 1510 cm^{-1}), Lorentzian (50, 1545 cm^{-1}), Gaussian (100, 1635 cm^{-1}), Gaussian (150, 1660 cm^{-1}), Lorentzian (25, 1745 cm^{-1}).

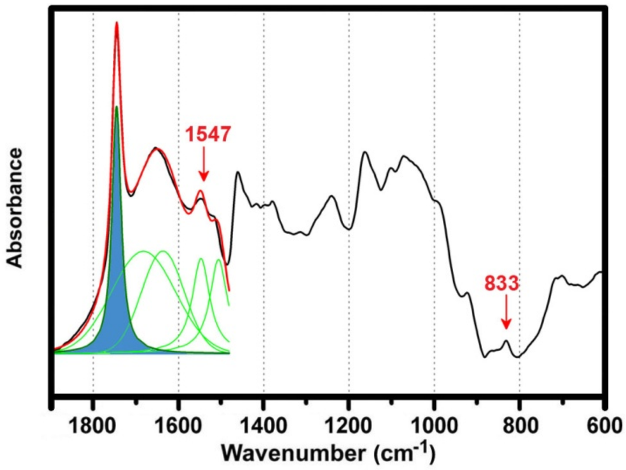

Fig. S1. Estimation of pollen lipid content for *Iris pallida* depicting bands for the normalization of transmission spectra (amide II band at 1547 cm^{-1}, and sporopollenin band at 833 cm^{-1} that was used as the secondary standard), spectral deconvolution (multipeak fitting of three Lorentzian and two Gaussian curves), and application of Beer–Lambert law, i.e. the linear correlation of absorbance (blue area under the curve with the peak center at ~1745 cm^{-1}) and lipid quantity.

Carbohydrate-to-protein ratio in pollen

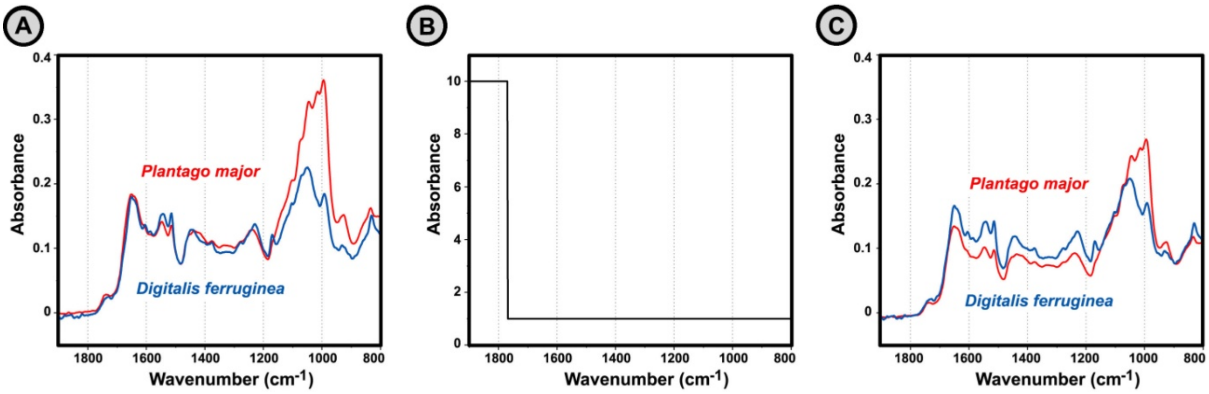

Fig. S2. (**A**) Reflectance IR spectra of *Plantago major* and *Digitalis ferruginea* (raw data). (**B**) Weighting vector for the MSC correction. (**C**) MSC corrected spectra of *Plantago major* and *Digitalis ferruginea*.

Pollen grain wall composition

Pollen grain has double-layered outer wall made of the cellulose-rich intine layer, and the highly resistant exine composed predominantly of a complex biopolymer sporopollenin. As a result of thick cellulose intine wall pollen grains of *Cupressaceae* family have unique and outlying spectral features (Fig. S3A). While *Cupressaceae* pollen grains have thick intine and thus increased relative amount of cellulose, pollens of *Pinaceae* family have a large hollow projection (saccus) from the central body of pollen grain composed only by the exine, resulting with increased relative amount of sporopollenin in the grains. For that reason the vibrational bands associated with the sporopollenin dominate to a larger extent in *Pinaceae* reflectance spectra than is the case with *Cupressaceae* spectra (Fig. S3D).

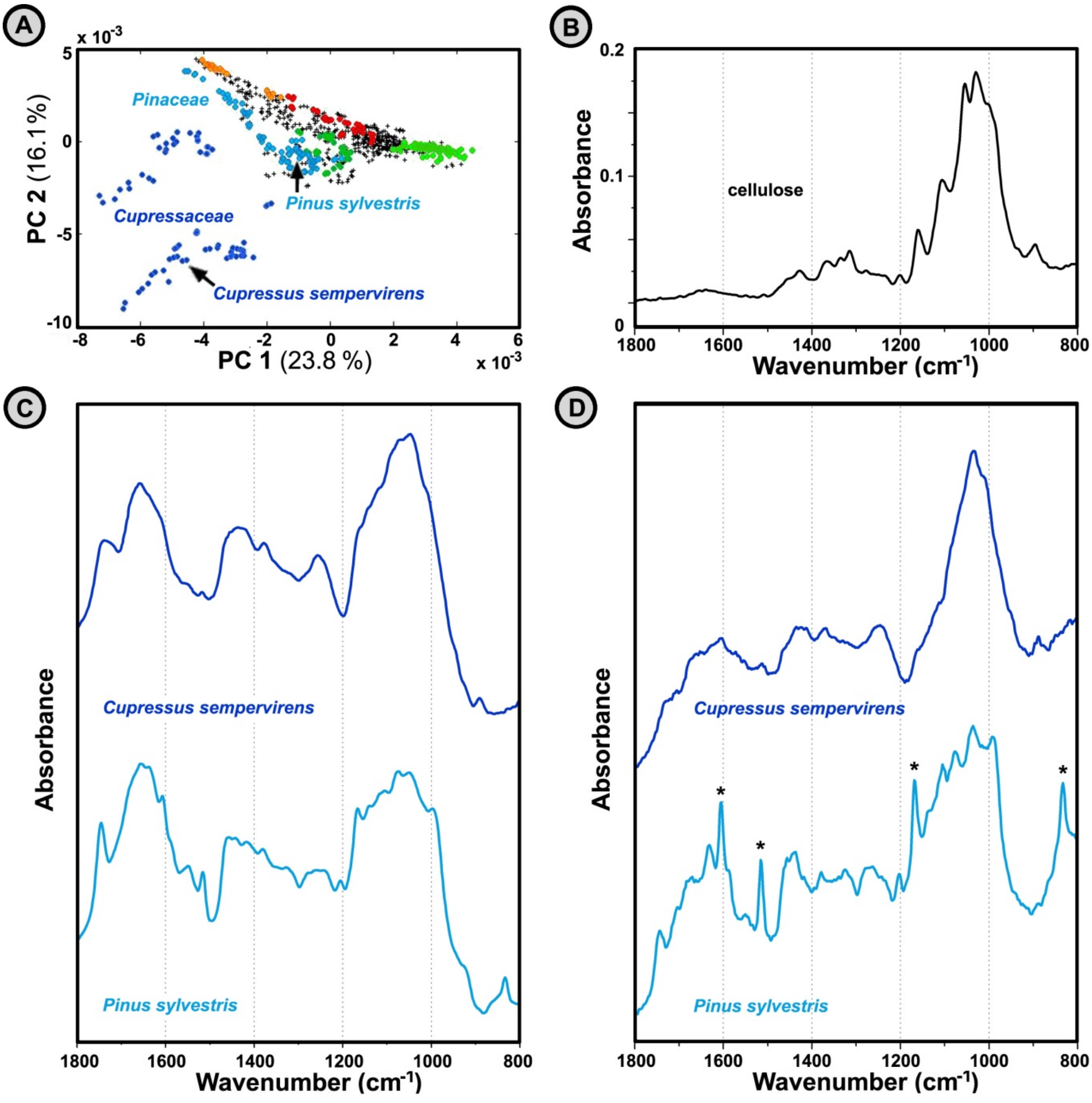

Fig. S3. (**A**) PCA plot of transmission IR spectral data set (300 species, three spectra per specie; second derivative and EMSC corrected spectra) with depiction of plant families: *Pinaceae* (light blue), *Cupressaceae* (dark blue), *Poaceae* (light green), *Cyperaceae* (dark green), *Betulaceae* (red), and *Juglandaceae* (orange). The percent variances for the first five PCs are 23.83, 16.13, 11.30, 6.11, and 5.24. (**B**) reflectance IR spectra of cellulose. EMSC corrected transmission (**C**) and reflectance (**D**) IR spectra of *Cupressus sempervirens* (Mediterranean cypress, *Pinales* order, *Cupressaceae* family) and *Pinus sylvestris* (Scots pine; *Pinales* order, *Pinaceae* family) pollen. For better viewing the spectra are offset; the vibrational bands associated with sporopollenin are denoted with asterisks.

Correlation between spectroscopic data and taxonomy

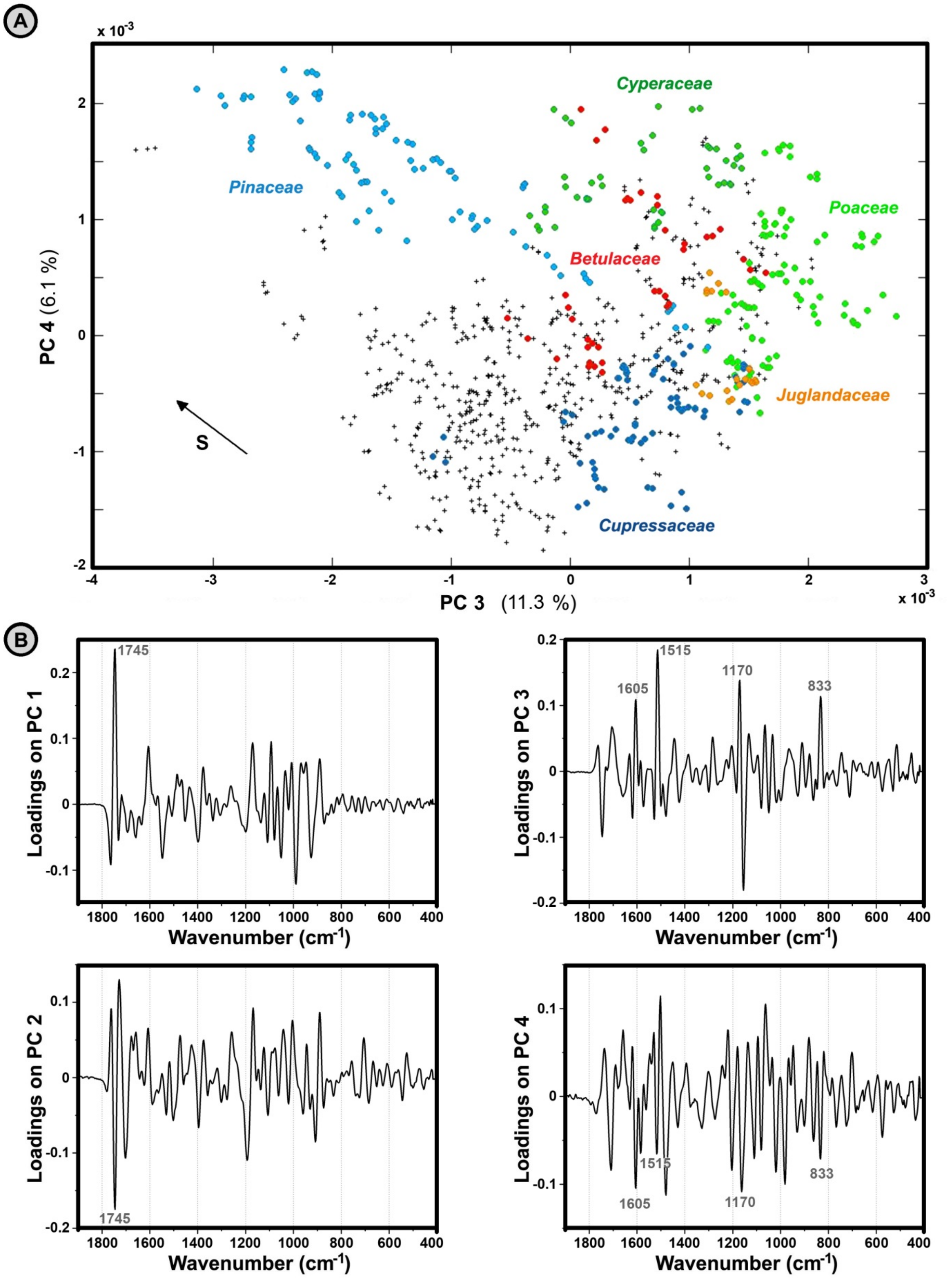

Fig. S4. (**A**) PCA plot of transmission IR spectral data set (300 species, three spectra per specie; second derivative and EMSC corrected spectra) with depiction of plant families: *Pinaceae* (light blue), *Cupressaceae* (dark blue), *Poaceae* (light green), *Cyperaceae* (dark green), *Betulaceae* (red), and *Juglandaceae* (orange). The percent variances for the first five PCs are 23.83, 16.13, 11.30, 6.11, and 5.24. (**B**) Loadings plot on the first four principal component of the PCA.

***Fagales* order pollen samples**

All pollen samples depicted in Fig. S5 were sampled in 2011 within the area of 100 m radius. The timeframe of the sampling was the following (expressed in weeks): *Corylus* (*W* denotes first week of March), *Alnus* (*W*), *Betula* (*W*+4), *Ostrya* (*W*+4), *Carpinus* (*W*+4), *Pterocarya* (*W*+4), *Juglans* (*W*+4 till W+6), *Quercus* (*W*+4 till *W*+7), *Fagus* (*W*+5) and *Carya* (*W*+10; i.e. third week of May).

Pollen samples depicted in Fig. S6 were sampled in 2010, in the first week of March. Five *Corylus avellana* samples (depicted in different shades of red) were collected from different individuals, all growing several meters apart on a same location.

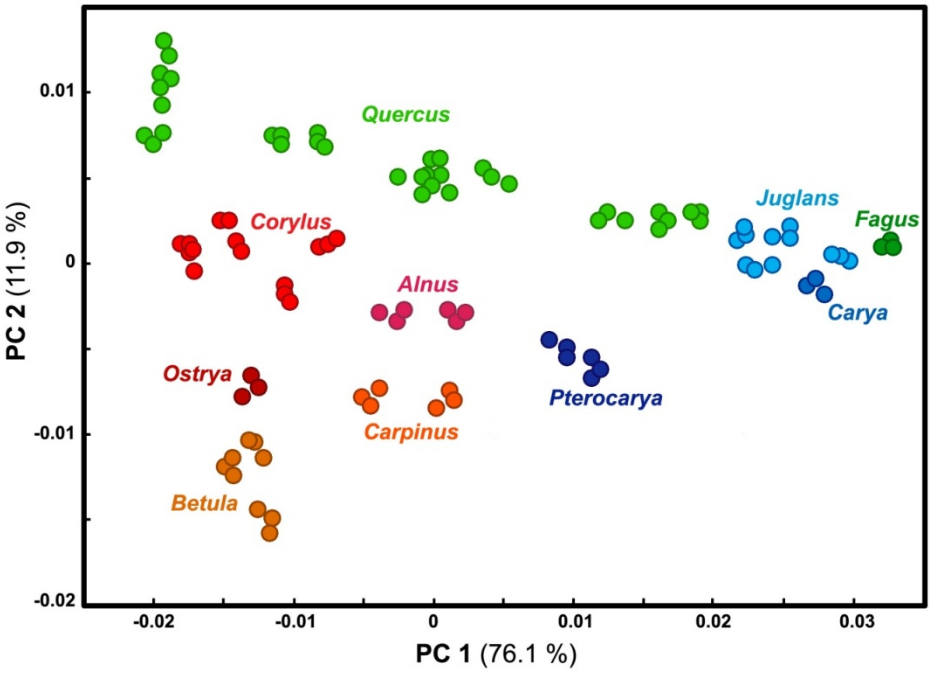

Fig. S5. (**A**) PCA plot of transmission IR spectral data set containing pollen samples of *Fagales* order in the 2011 pollination season (33 species, three spectra per specie; first derivative and EMSC corrected spectra) with depiction of plant families: *Betulaceae* (red: *Corylus* (hazel), *Alnus* (alder), *Betula* (birch), *Carpinus* (hornbeam) and *Ostrya* (hop-hornbeam)), *Fagaceae* (green: *Fagus* (beech) and *Quercus* (oak)), and *Juglandaceae* (blue: *Juglans* (walnut), *Pterocarya* (wingnut) and *Carya* (hickory)). The percent variances for the first five PCs are 76.05, 11.85, 3.78, 2.75, and 1.24.

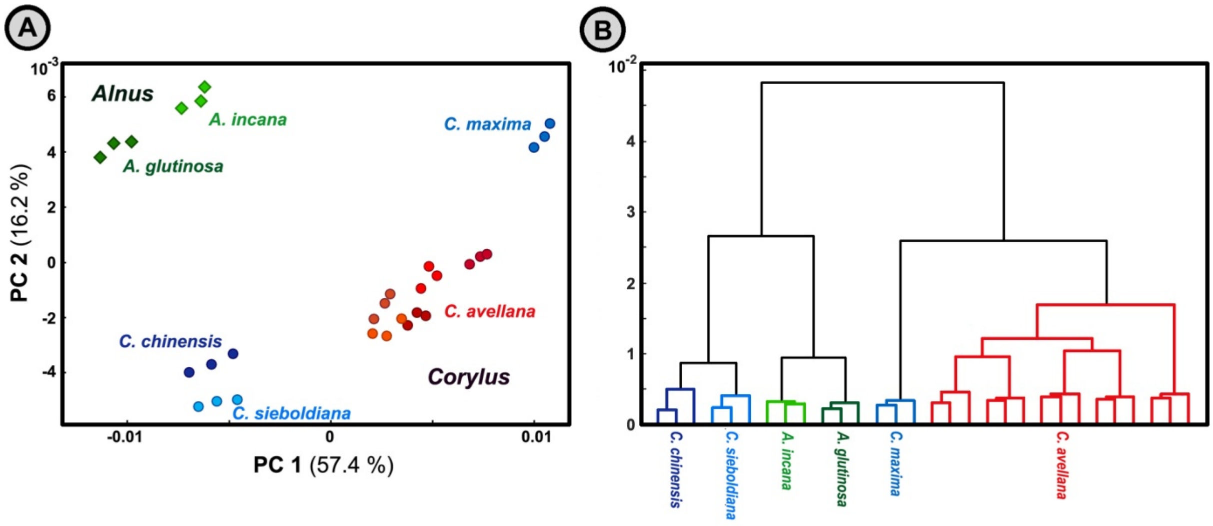

Fig. S6. Reflectance IR spectral data set containing pollen samples of *Betulacea* family in the 2010 pollination season (10 samples, three spectra per sample; first derivative and EMSC corrected spectra) with depiction of plant genera and species (two *Alnus* and four *Corylus* species). (**A**) PCA plot of spectral data; The percent variances for the first five PCs are 57.39, 16.19, 11.33, 6.06, and 1.93. (**B**) HCA dendrogram.

Triglycerides content of pollen

The spectral variability caused by the specific biochemicals can be extracted from the rest of the data (Fig. S7). That way, the influence of specific biochemical components on spectral variability, such as triglycerides, can be evaluated.

The data matrix containing complete set of transmission spectra was deflated by using either 1) the PC 1q vector (the first principal component obtained by PCA on quercus subset; i.e. set of transmission spectra obtained by measuring pollen samples of *Quercus* genus), or 2) reflectance spectra of tristearin (Fig. S7D). For the deflation the data matrix was centred while the vectors were normalized. The PC 1q vector accounts for the maximal spectral variance in congenital pollen samples of *Quercus* genus. Since both deflation procedures resulted with the similar reduced data sets, as seen by the PCA plots of this sets (Figs. S7B and C), it is clear that congenital differentiation of *Quercus* pollen, as well as *Pinus* and *Iris* pollen, can be based on the relative content of triglycerides. The impact of triglycerides on spectral variance can be more directly estimated from spectral differences of congenital pollen samples (Fig. S7E)

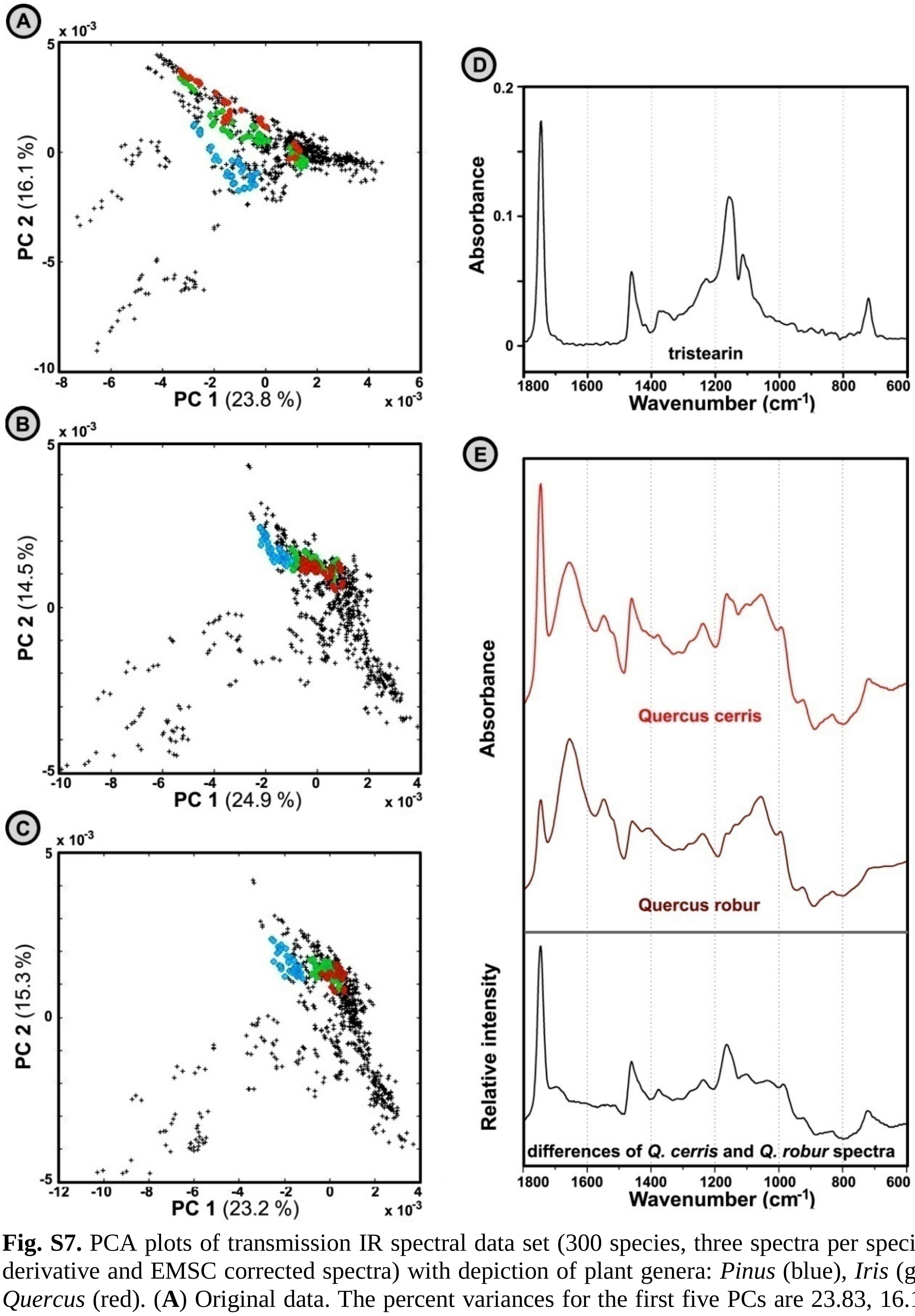

Fig. S7. PCA plots of transmission IR spectral data set (300 species, three spectra per specie; second derivative and EMSC corrected spectra) with depiction of plant genera: *Pinus* (blue), *Iris* (green) and *Quercus* (red). (**A**) Original data. The percent variances for the first five PCs are 23.83, 16.13, 11.30, 6.11, and 5.24. (**B**) Modelling spectral contribution of triglycerides, approximated by tristearin spectrum. The percent variances for the first five PCs are 24.87, 14.48, 9.40, 7.17, and 5.91. (**C**) Modelling spectral contribution of PC 1q obtained by PCA on quercus subset. The percent variances for the first five PCs are 23.19, 15.27, 7.43, 6.22, and 5.71. Note the similarity between figures **B** and **C**: It is clear that triglyceride content is responsible for the majority of congenital spectral variations. (**D**) reflectance IR spectra of tristearin (amorphous phase). (**E)** Transmission IR spectra of *Quercus cerris* (Turkey oak) and *Quercus robur* (pedunculate oak) pollen, and spectral differences of the two transmission spectra. For better viewing the spectra are offset.

Table S1. List of analyzed pollen taxa (part I): eudicots.

ORDER	FAMILY	GENUS	SPECIES	COMMON NAME
Fagales	*Betulaceae*	*Alnus*	*A. incana*	Grey Alder
			A. glutinosa	Black Alder
		Corylus	*C. maxima*	Filbert
			C. avellana	Common Hazel
			C. chinensis	Chinese Hazel
			C. colurna	Turkish Hazel
			C. sieboldiana	Asian Beaked Hazel
		Ostrya	*O. carpinifolia*	European Hop-hornbeam
		Betula	*B. ermanii*	Erman's Birch
			B. pendula	Silver Birch
			B. papyrifera	Paper Birch
		Carpinus	*C. orientalis*	Oriental Hornbeam
			C. betulus	European Hornbeam
	Fagaceae	*Quercus*	*Q. robur*	Pedunculate Oak
			Q. coccinea	Scarlet Oak
			Q. rubra	Northern Red Oak
			Q. petreae	Sessile Oak
			Q. ilex	Holly Oak
			Q. coccifera	Kermes Oak
			Q. faginea	Portuguese Oak
			Q. cerris	Turkey Oak
			Q. pubescens	Downy Oak
			Q. libani	Lebanon Oak
			Q. shumardii	Shumard's Oak
			Q. frainetto	Hungarian Oak
		Fagus	*F. sylvatica*	European Beech
	Juglandaceae	*Juglans*	*J. regia*	Persian Walnut
			J. ailanthifolia	Japanese Walnut
			J. mandshurica	Manchurian Walnut
			J. nigra	Eastern Black Walnut
		Carya	*C. illinoinensis*	Pecan Hickory
		Pterocarya	*P. stenoptera*	Chinese Wingnut
			P. fraxinifolia	Caucasian Wingnut
Malpighiales	*Salicaceae*	*Salix*	*S. babylonica*	Peking Willow
			S. alba	White Willow
			S. caprea	Goat Willow
		Populus	*P. nigra* *	Black Poplar
			P. alba *	White Poplar
	Euphorbiaceae	*Ricinus*	*R. communis*	Castor Bean
Proteales	*Platanaceae*	*Platanus*	*P. x hispanica*	Hybrid (London) Plane
			P. orientalis	Oriental Plane
Lamiales	*Oleaceae*	*Fraxinus*	*F. excelsior*	European Ash
			F. pennsylvanica	Red Ash
			F. ornus	Manna Ash
			F. chinensis	Chinese Ash
		Olea	*O. europeae*	Olive
	Plantaginaceae	*Plantago*	*P. lanceolata*	Ribwort Plantain
			P. media	Hoary Plantain
			P. major	Greater Plantain
		Digitalis	*D. purpurea*	Common Foxglove
			D. grandiflora	Big-flowered Foxglove
			D. lutea	Yellow Foxglove
			D. ferruginea	Rusty Foxglove
	Scrophulariacea	*Paulownia*	*P. tomentosa*	Empress Tree
	Acanthaceae	*Acanthus*	*A. balcanicus*	Balcan Bear's Breeches
Saxifragales	*Altingiaceae*	*Liquidambar*	*L. orientalis*	Oriental Sweetgum
			L. styraciflua	American Sweetgum
	Paeoniaceae	*Paeonia*	*P. mascula*	Wild Peony
			P. officinalis	European Peony

Note on Table S1: Species denoted with asterisks were measured in transmission mode only.

Table S1. List of analyzed pollen taxa (part II): eudicots.

ORDER	FAMILY	GENUS	SPECIES	COMMON NAME
Caryophyllales	*Polygonaceae*	*Rumex*	*R. acetosa*	Common Sorrel
			R. longifolius	Dooryard Dock
			R. patientia	Patience Dock
			R. aquaticus	Western Dock
			R. alpinus	Alpine Dock
	Portulacaceae	*Cistanthe*	*C. grandiflora*	Big-flowered Pussypaws
Sapindales	*Sapindaceae*	*Aesculus*	*A. hippocastanum*	Common Horse Chestnut
			A. flava	Yellow Buckeye
		Acer	*A. opalus*	Italian Maple
			A. negundo *	Boxelder Maple
			A. cissifolium	Vineleaf Maple
	Anacardiaceae	*Pistacia*	*P. lentiscus*	Mastic Tree
			P. terebinthus	Terebinth
	Rutaceae	*Dictamnus*	*D. albus*	White Dittany
Dipsacales	*Adoxaceae*	*Sambucus*	*S. nigra*	Black Elder
		Viburnum	*V. rhytidophyllum*	Leatherleaf Viburnum
			V. carlesii	Korean Spice Viburnum
			V. lantana	Wayfaring Tree
			V. opalus	Guelder Rose
	Dipsacaceae	*Scabiosa*	*S. caucasica*	Caucasian Scabious
		Cephalaria	*C. gigantea*	Tatarian Cephalaria
Asterales	*Asteraceae*	*Ambrosia*	*A. artemisifolia*	Common Ragweed
		Taraxacum	*T. officinale*	Common Dandelion
		Artemisia	*A. absinthium*	Absinthe Wormwood
			A. dracunculus	Tarragon
			A. vulgaris	Common Wormwood
	Campanulaceae	*Platycodon*	*P. grandiflorus*	Chinese Bellflower
		Campanula	*C. persicifolia*	Peach-leaved Bellflower
Rosales	*Rosaceae*	*Prunus*	*P. cerasifera*	Cherry Plum
			P. padus	Bird Cherry
			P. laurocerasus	Cherry Laurel
		Rosa	*R. pendulina*	Alpine Rose
			R. canina	Dog Rose
			R. dumetorum	Corymb Rose
		Rubus	*R. fruticosus*	Common Blackberry
		Mespilus	*M. germanica*	Common Medlar
		Sanguisorba	*S. minor*	Salad Burnet
		Potentilla	*P. atrosanguinea*	Ruby Cinquefoil
		Geum	*G. x heldreichii*	Copper Avens
		Fragaria	*F. moschata*	Musk Strawberry
		Filipendula	*F. vulgaris*	Fern-leaf Dropwort
	Urticaceae	*Parietaria*	*P. officinalis*	Upright Pellitory
		Urtica	*U. pilulifera*	Roman Nettle
			U. dioica	Stinging Nettle
Buxales	*Buxaceae*	*Buxus*	*B. sempervirens*	Common Box
			B. microphylla	Japanese Box
Malvales	*Malvaceae*	*Abutilon*	*A. grandifolium*	Hairy Indian Mallow
		Kitaibelia	*K. vitifolia*	Vine-leaved Kitaibelia
		Althaea	*A. officinalis*	Common Marshmallow
			A. cannabina	Palm-leaf Marshmallow
Geraniales	*Geraniaceae*	*Geranium*	*G. phaeum*	Dusky Cranesbill
Fabales	*Fabaceae*	*Bauhinia*	*B. purpurea*	Purple Orchid Tree
Brassicales	*Brassicaceae*	*Bunias*	*B. orientalis* *	Turkish Wartycabbage
	Tropaeolaceae	*Tropaeolum*	*T. majus*	Garden Nasturtium
Cucurbitales	*Cucurbitaceae*	*Ecballium*	*E. elaterium*	Squirting Cucumber

Note on Table S1: Species denoted with asterisks were measured in transmission mode only.

Table S1. List of analyzed pollen taxa (part III): eudicots and magnoliids.

ORDER	FAMILY	GENUS	SPECIES	COMMON NAME
Solanales	*Solanaceae*	*Nicandra*	*N. physalodes*	Apple of Peru
		Nicotiana	*N. alata*	Winged Tobacco
			N. tabacum	Common Tobacco
		Datura	*D. inoxia*	Downy Thorn-apple
		Brugmansia	*B. suaveolens*	White Angel's Trumpet
Ranunculales	*Ranunculaceae*	*Pulsatilla*	*P. montana*	Mountain Pasque Flower
		Aquilegia	*A. nigricans*	Bulgarian Columbine
			A. vulgaris	European Columbine
			A. chrysantha	Golden Columbine
			A. fragrans	Fragrant Columbine
		Anemone	*A. coronaria*	Poppy Anemone
			A. sylvestris	Snowdrop Windflower
		Caltha	*C. palustris*	Marsh Marigold
		Ranunculus	*R. repens*	Creeping Buttercup
			R. acris	Meadow Buttercup Žabnjak
			R. lanuginosus	Woolly Buttercup
		Clematis	*C. recta*	Erect Clematis
		Nigella	*N. damascena*	Love-in-a-Mist
	Papaveraceae	*Papaver*	*P. lapponicum*	Lapland Poppy
			P. orientale	Oriental Poppy
			P. nudicaule	Iceland Poppy
			P. rhoeas	Field Poppy
		Glaucium	*G. flavum*	Yellow Hornpoppy
		Eschscholzia	*E. californica*	California Poppy
		Chelidonium	*C. majus*	Greater Celandine
Magnoliales	*Magnoliaceae*	*Magnolia*	*M. x sonlangiana*	Saucer Magnolia
		Liriodendron	*L. tulipifera*	American Tulip Tree

Table S1. List of analyzed pollen taxa (part IV): monocots.

ORDER	FAMILY	GENUS	SPECIES	COMMON NAME
Poales	*Cyperaceae*	*Holoschoenus*	*H. romanus*	Round-headed Club-rush
		Carex	*C. dipsacea*	New Zealand Sedge
			C. pendula	Pendulous Sedge
			C. muskingumensis	Muskingum Sedge
			C. flacca	Blue Sedge
			C. divisa	Divided Sedge
			C. ferruginea	Rusty Sedge
			C. morrowii	Variegata Sedge
			C. riparia	Great Pond-Sedge
			C. ornithopoda	Birdsfoot Sedge
			C. grayi	Gray's Sedge
			C. sylvatica	Forest Sedge
			C. ovalis	Eggbract Sedge
		Cladium	*C. mariscus*	Sawtooth Sedge
		Eleocharis	*E. palustris*	Common Spikerush
	Poaceae	*Secale*	*S. cereale*	Rye
		Zea	*Z. mays*	Maize
		Alopecurus	*A. pratensis*	Meadow Foxtail
		Brachypodium	*B. retusum*	Mediterranean False-brome
			B. pinnatum	Heath False-brome
		Piptatherum	*P. miliaceum*	Smilograss
		Festuca	*F. filiformis*	Fine-Leaved Sheep's Fescue
			F. ovina	Sheep Fescue
			F. pratensis	Meadow Fescue
			F. drymeja	Mountain Fescue
			F. amethystina	Tufted Fescue
			F. arvernensis	Field Fescue
			F. heterophylla	Various-leaved Fescue
		Melica	*M. ciliata* *	Hairy Melic
			M. altissima	Siberian Melic
			M. nutans	Mountain Melic
		Poa	*P. badensis*	Baden's Bluegrass
			P. pratensis	Smooth Meadow-grass
			P. nemoralis	Wood Meadow-Grass
			P. compressa	Flattened Meadow-grass
		Anthoxanthum	*A. odoratum*	Sweet Vernal Grass
		Arrhenatherum	*A. elatius*	Tall Oat-grass
		Sesleria	*S. tenuifolia*	Thin-leaved Moor Grass
			S. nitida	Nest Moor Grass
		Dactylis	*D. glomerata*	Orchard Grass
		Pennisetum	*P. orientale* *	Oriental Fountain Grass
		Bromus	*B. erectus*	Erect Brome
		Briza	*B. media*	Common Quaking Grass
		Lolium	*L. perenne*	Perennial Ryegrass
		Holcus	*H. lanatus*	Common Velvet Grass
		Phalaris	*P. canariensis*	Phalaris canariensis
		Coix	*C. lacryma-jobi*	Job's Tears
	Juncaceae	*Luzula*	*L. luzuloides*	White Wood-Rush
			L. sylvatica	Great Wood-Rush
	Typhaceae	*Typha*	*T. latifolia*	Broadleaf Cattail
Arales	*Acoraceae*	*Acorus*	*A. calamus*	Sweet Flag
Zingiberales	*Cannaceae*	*Canna*	*C. orchiodes*	Orchid Canna
Arecales	*Arecaceae*	*Chamaerops*	*C. humilis* (stak)	Mediterranean Fan Palm

Note on Table S1: Species denoted with asterisks were measured in transmission mode only.

Table S1. List of analyzed pollen taxa (part V): monocots.

ORDER	FAMILY	GENUS	SPECIES	COMMON NAME
Asparagales	*Hyacinthaceae*	*Hyacinthus*	*H. orientalis*	Garden Hyacinth
		Hyacinthoides	*H. hispanica*	Spanish Bluebell
	Iridaceae	*Iris*	*I. illyrica*	Illyrian Iris
			I. pseudacorus	Yellow Iris
			I. japonica	Japanese Iris
			I. germanica	German Iris
			I. sikkimensis	Sikkim Iris
			I. versicolor	Larger Blue Flag
			I. pseudopallida	South Adriatic Iris
			I. sibirica	Siberian Iris
			I. sanguinea	Blood Iris
			I. graminea	Grass-Leaved Iris
			I. spuria	Blue Iris
			I. crocea	Crosier Iris
			I. unguicularis	Algerian Iris
			I. aphylla	Stool Iris
			I. bulleyana	Bulley's Iris
			I. pallida	Dalmatian Iris
			I. bucharica	Corn Leaf Iris
			I. orientalis	Yellow-banded Iris
		Gladiolus	*G. italicus*	Italian Gladiolus
	Asphodelaceae	*Asphodelus*	*A. aestivus*	Summer Asphodel
	Xanthorrhoeacea	*Hemerocallis*	*H. lilioasphodelus*	Yellow Daylily
			H. minor	Dwarf Daylily
			H. citrina	Citron Daylily
			H. esculenta	Japanese Daylily
		Aloe	*A. grandidentata*	Dwarf Soap Aloe
			A. capitata	Capitate Aloe
			A. striata	Coral Aloe
		Kniphofia	*K. angustifolia*	Thin-leaved Tritoma
			K. uvaria	Red Tritoma
			K. triangularis	Triangle Tritoma
		Gasteria	*G. obliqua*	Bicolor Gasteria
		Asphodeline	*A. lutea*	Yellow Asphodel
	Asparagaceae	*Hosta*	*H. plantaginea*	Fragrant Hosta
			H. sieboldiana	Sieboldiana Hosta
			H. kikutii	Rock Hosta
Liliales	*Liliaceae*	*Fritillaria*	*F. imperialis*	Crown Imperial
		Lilium	*L. bulbiferum*	Orange Lily
			L. carniolicum	Carniolan Lily
			L. pumilum	Siberian Lily
			L. martagon	Martagon Lily
			L. regale	Regal Lily
			L. monadelphum	Caucasian Lily
			L. candidum	Madonna Lily
			L. japonicum	Bamboo Lily
			L. henryi	Tiger Lily
			L. henryi	Tiger Lily

Table S1. List of analyzed pollen taxa (part VI): conifers and ginkos.

ORDER	FAMILY	GENUS	SPECIES	COMMON NAME
Pinales	*Cupressaceae*	*Platycladus*	*P. orientalis*	Chinese Arborvitae
		Thujopsis	*T. dolabrata*	Thujopsis
		Chamaecyparis	*C. lawsoniana*	Lawson's Cypress
			C. obtusa	Hinoki Cypress
			C. pisifera	Sawara Cypress
		Juniperus	*J. chinensis*	Chinese Juniper
			J. virginiana	Eastern Juniper
			J. sabina	Savin Juniper
			J. phoenicea	Phoenicean Juniper
			J. communis	Common Juniper
			J. oxycedrus	Prickly Juniper
			J. excelsa	Greek Juniper
		Calocedrus	*C. decurrens*	California Incense-cedar
		Cupressus	*C. sempervirens*	Mediterranean Cypress
			C. x leylandii	Leyland Cypress
			C. lusitanica	Mexican Cypress
			C. sargentii	Sargent Cypress
		Cunninghamia	*C. lancelota*	China Fir
		Cryptomeria	*C. japonica*	Japanese Cedar
		Thuja	*T. occidentalis*	Eastern Arborvitae
			T. standishii	Japanese Thuja
		Taxodium	*T. distichum*	Bald Cypress
		Sequoia	*S. sempervirens*	Coast Redwood
		Metasequoia	*M. glyptostroboides*	Dawn Redwood
	Taxaceae	*Taxus*	*T. baccata*	European Yew
			T. cuspidata	Japanese Yew
	Cephalotaxaceae	*Cephalotaxus*	*C. harringtonia*	Japanese Plum Yew
		Torreya	*T. californica*	California Torreya
	Pinaceae	*Tsuga*	*T. canadensis*	Eastern Hemlock
		Cedrus	*C. atlantica*	Atlas Cedar
			C. deodara	Deodar Cedar
		Abies	*A. koreana*	Korean Fir
			A. pinsapo	Spanish Fir
			A. alba	Silver Fir
			A. cephalonica	Greek Fir
		Picea	*P. abies*	Norway Spruce
			P. asperata	Dragon Spruce
			P. omorika	Serbian Spruce
			P. chihuahuana	Chihuahua Spruce
			P. orientalis	Caucasian Spruce
			P. smithiana	Morinda Spruce
			P. pungens	Blue Spruce
		Pinus	*P. pinea*	Stone Pine
			P. mugo	Mountain Pine
			P. sylvestris	Scots Pine
			P. tabuliformis	Chinese Red Pine
			P. banksiana	Jack Pine
			P. pinaster	Maritime Pine
			P. densiflora	Japanese Red Pine
			P. nigra	European Black Pine
			P. ponderosa	Ponderosa Pine
			P. resinosa	Red Pine
			P. wallichiana	Himalayan pine
			P. bungeana	Lacebark Pine
			P. peuce	Macedonian Pine
			P. strobus	Eastern White Pine
			P. heldreichii	Bosnian Pine
	Podocarpaceae	*Podocarpus*	*P. neriifolius*	Brown Pine
Ginkgoales	*Ginkgoaceae*	*Ginkgo*	*G. biloba*	Ginkgo
